# O3-6 The Healthy School Start Plus Study - A parental support programme to promote healthy behaviours and prevent childhood obesity in disadvantaged areas

**DOI:** 10.1093/eurpub/ckac094.022

**Published:** 2022-08-29

**Authors:** Mahnoush Etminan Malek, Åsa Norman, Liselotte Schäfer Elinder, Emma Patterson, Gisela Nyberg

**Affiliations:** 1 Global Public Health, Karolinska Institutet, Stockholm, Sweden; 2 Centre for Epidemiology and Community Medicine, Region Stockholm, Stockholm, Sweden; 3 The Swedish School of Sport and Health Sciences, The Swedish School of Sport and Health Sciences, Stockholm, Sweden

**Keywords:** Diet, Healthy behaviours, Socioeconomic position, Elementary school, Parental involvement

## Abstract

**Background:**

The rise in childhood overweight and obesity worldwide demands effective health promotion and obesity prevention programmes, especially targeting socially disadvantaged areas. The aim of this study is to examine the effectiveness of a revised parental support programme, promoting physical activity and a healthy diet, and on preventing overweight and obesity among children in disadvantaged areas.

**Methods:**

The effectiveness of this programme will be compared to standard school routines in a parallel group cluster randomised controlled trial. The 6-month programme included: 1) A health information brochure; 2) School nurses conducting motivational interviewing with parents; 3) Classroom activities and home assignments for children; 4) A self-test for type-2 diabetes risk for parents. Seventeen schools were enrolled including 352 six-year-old children (155 intervention/197 control). Physical activity and sedentary time were measured by accelerometry. Dietary intake was assessed by a newly developed mobile phone-based photo method. Weight and height were measured by trained researchers. All outcomes were measured at baseline and at 6 and 18 months post baseline. Parental level of education was self-reported, and the highest level achieved by either parent was used as an indicator of socioeconomic position (SEP). A mixed-effect regression analysis will be performed to evaluate the effectiveness of the programme.

**Results:**

After the intervention, when adjusting for sex and parental education, the intervention group showed 6.4 mins more moderate to vigorous physical activity (MVPA) during weekdays than the control group (p = 0.03). No significant effect on MVPA was detected during weekends (p = 0.47). Further, no significant effect was detected on time spent sedentary during weekdays (p = 0.12) nor during weekends (p = 0.78).

According to IOTF classifications, 9.6% of the children had obesity, 16.4% overweight, 4.4% underweight and 69.6% normal weight. Results on changes in BMI, and dietary intake at 6 months will be presented.

**Conclusions:**

The results from the evaluation of this parental support programme will add to the knowledge and advance intervention and implementation research in the Swedish/Nordic context relating to prevention of overweight and obesity in childhood.

